# Differential diagnosis between patients with probable Alzheimer’s disease, Parkinson’s disease dementia, or dementia with Lewy bodies and frontotemporal dementia, behavioral variant, using quantitative electroencephalographic features

**DOI:** 10.1007/s00702-017-1699-6

**Published:** 2017-02-27

**Authors:** Heinrich Garn, Carmina Coronel, Markus Waser, Georg Caravias, Gerhard Ransmayr

**Affiliations:** 1 AIT Austrian Institute of Technology GmbH, Donau-City-Straße 1, 1220 Vienna, Austria; 2 Brunnenweg 2, 4810 Gmunden, Austria; 3 Department of Neurology 2 at the MedCampus III of the Kepler University Hospital, Krankenhausstraße 9, 4021 Linz, Austria

**Keywords:** Alzheimer’s disease, Dementia with Lewy bodies, Frontotemporal dementia, Parkinson’s disease dementia, Quantitative electroencephalogram

## Abstract

The objective of this work was to develop and evaluate a classifier for differentiating probable Alzheimer’s disease (AD) from Parkinson’s disease dementia (PDD) or dementia with Lewy bodies (DLB) and from frontotemporal dementia, behavioral variant (bvFTD) based on quantitative electroencephalography (QEEG). We compared 25 QEEG features in 61 dementia patients (20 patients with probable AD, 20 patients with PDD or probable DLB (DLBPD), and 21 patients with bvFTD). Support vector machine classifiers were trained to distinguish among the three groups. Out of the 25 features, 23 turned out to be significantly different between AD and DLBPD, 17 for AD versus bvFTD, and 12 for bvFTD versus DLBPD. Using leave-one-out cross validation, the classification achieved an accuracy, sensitivity, and specificity of 100% using only the QEEG features Granger causality and the ratio of theta and beta1 band powers. These results indicate that classifiers trained with selected QEEG features can provide a valuable input in distinguishing among AD, DLB or PDD, and bvFTD patients. In this study with 61 patients, no misclassifications occurred. Therefore, further studies should investigate the potential of this method to be applied not only on group level but also in diagnostic support for individual subjects.

## Introduction

Alzheimer´s disease (AD), the most frequent degenerative dementia, is clinically characterized by progressive impairment of episodic memory and other cognitive domains (e.g., language, reasoning, visuospatial functions, managing complex tasks, daily routine, and behavioral abnormalities). AD is typically associated with the dysfunction of the temporal, parietal, and also occipital lobes and atrophy of the hippocampus.

In Parkinson’s disease, up to 85% of patients develop Parkinson’s disease dementia (PDD) in the course of their motor disease (Dubois et al. 2007; Emre et al. 2007). PDD often appears in combination with psychotic features, depression, and marked impairment of axial motor functions. However, some patients also develop dementia, psychotic episodes, fluctuations of vigilance and cognition, and parkinsonian motor features combined with autonomic symptoms and REM sleep behavior disorder within a short period of time, or dementia may precede levodopa-responsive parkinsonian motor symptoms. The term dementia with Lewy bodies (DLB) has been coined (McKeith et al. 2005) to describe this condition; however, clinical similarities of PDD and DLB have been emphasized for clinical practice (McKeith et al. McKeith et al. 2005).

Frontotemporal dementia (FTD) is caused by frontotemporal lobar degeneration. The differential diagnosis of FTD may be difficult due to the heterogeneity of partly overlapping the symptoms. Three groups are distinguished (motor, language, and behavior), where the behavioral variant FTD (bvFTD) is characterized by changes in social behavior and conduct, with loss of social awareness, apathy, hyperorality and dietary changes, and poor impulse control combined with deficits in executive tasks (Rascovsky et al. 2011). FTD accounts for up to 20% of young-onset dementia cases. Diagnostic criteria exist (Rascovsky and Grossmann 2013), but the disease remains poorly recognized yet.

Clinicopathological studies suggest differences, but also a continuum between and neuropathological overlaps of the three disorders; however, specific diagnosis is warranted for prognosis and treatment. Difficulties of differential diagnosis have been discussed (Bonanni et al. 2008; Li et al. 2016), concluding that the clinical symptoms can vary largely in that they may variably appear during the course of the disease. There are no specific biological markers for these diseases. Differential diagnosis may be complex and expensive [magnetic resonance (MR) imaging (MRI), dopamine transporter (FP-Cit) single photon emission computed tomography (SPECT), FDG or amyloid positron emission tomography (PET), cerebrospinal fluid examination, repeated clinical and neuropsychological evaluation, and genetic testing]. Moreover, positivity in amyloid PET, the most recent advanced diagnostic tool, is not AD specific and may also be found in DLB and FTD (Ossenkoppele et al. 2015).

Consequently, there is an ongoing need for diagnostic improvement. Electroencephalography (EEG) is cost-effective and available as the standard equipment in most primary, secondary, and tertiary neurological and psychiatric referral centers and in neurological practice. Significant correlations between various features of quantitative EEG (QEEG) and the severity of AD have already been demonstrated (Garn et al. 2014, 2015). A slowing of the frequency spectrum to variable extent is a characteristic feature of degenerative dementias, such as AD or DLB (Li et al. 2016; Jeong 2004; Caso et al. 2012; Lindau et al. 2003). We hypothesized that, based on the previous findings, frequency spectrum analysis together with more complex parameters and statistical evaluation of QEEG could be applied in the differential diagnosis of AD, DLB, PDD, and bvFTD.

Only one study has investigated the possibility of differential diagnosis between three different forms of dementia by QEEG so far. Snaedal et al. (2012) used a database of EEGs from seven different groups of subjects with cognitive impairment and dementia. A classifier was created for each possible pair of groups using statistical pattern recognition. Accuracies of 91% (DLBPD-AD), 93% (DLBPD-FTD), and 88% (AD-FTD) were achieved for classifications between groups.

Differentiation between AD and PDD or probable DLB (DLBPD) was reported in (Bonanni et al. 2008; Fonseca et al. 2013; Babiloni et al. 2011; Kai et al. 2005), but no quantitative classification results were given. Andersson et al (2008) evaluated EEG variability in dementia with DLB and AD. Their features could distinguish DLB patients from AD patients and controls with areas under the ROC curves ranging between 0.75 and 0.80 and 0.91 and 0.97, respectively. For the differentiation between AD and FTD, three studies can be found: Caso et al. (2012) used a single subject analysis employing 12 spectral parameters and achieved 48.72% sensitivity and 85% specificity. Lindau et al. (2003) performed a logistic regression model. A classification accuracy of 93.3% was achieved using δ and θ activities, visuospatial ability, and episodic memory. Nishida et al. (2011) found significant differences in the relative global field power in the frequency band 12.5–18 Hz but did not give quantitative results.

The purpose of our study was to identify and evaluate QEEG features that would help distinguishing patients with AD, DLBPD, and bvFTD. These features were derived from multi-channel EEG recordings made in resting state in both time and frequency domains. The goal was to generate quantitative, statistically significant criteria that could be applied to patients in routine assessments in the future. The method addresses subjects with clear cognitive deficiencies, evident from clinical appearance, and neuropsychological test scores. Consequently, the study included no healthy controls.

## Methods

### Subjects

Patients were recruited at the Department of Neurology 2 of the Kepler University Hospital Linz. All subjects had a history and presented evidence of progressive cognitive decline from previous levels of performance interfering with abilities to function in usual activities, reported by themselves or by external informants, mostly spouses, and assessed clinically and neuropsychologically. Routine blood laboratory parameters, including serum vitamin B12 and folate serum levels, thyroid gland parameters, HIV, and syphilis serology, were assessed to rule out unknown metabolic and infectious diseases. The Mini Mental State Examination (MMSE, Folstein et al. 1975), the CERADplus neuropsychological battery (Schmid et al. 2014), the Clock Drawing Test (Sunderland et al. 1989), and the Frontal Assessment Battery (Dubois et al. 2000) were used for cognitive testing. Psychiatric disorders, substance abuse, medication, or other hypothesized or proven diagnoses that could otherwise explain the cognitive decline were ruled out as well as delirium at the time of diagnosis, neuropsychological testing, and EEG registration. All patients with probable Alzheimer dementia (AD) and frontotemporal dementia, behavioral variant (FTDbv, clinical criteria, and diagnosis, see in the following), had a 1.5 or 3 Tesla MRI of the brain (T1, T2, FLAIR, DTI, and T2* sequences). Patients with Parkinson’s disease dementia (PDD) and dementia with Lewy Bodies (DLB) had either cerebral MRI (1.5 or 3 Tesla, *N* = 12) or CT (*N* = 8).

Patients with probable AD (*N* = 20) and FTDbv (*N* = 21) participated in prospective longitudinal studies of the Austrian Alzheimer Society (PRODEM) and of the Kepler University Hospital Linz (FTLA Study), respectively, which had been reviewed by the local ethical committee (Ethikkommission des Landes Oberösterreich, approval number 254). Patients and informants, mostly family members, were included in the study after written informed consent. Patients participating at the PRODEM and FTLA studies were neuropsychologically tested but also evaluated for neuropsychiatric and behavioral symptoms using the neuropsychiatric Inventory (NPI, Cummings 1994), activities of daily living, and caregiving. Patients in these studies were followed at 6-months to 1-year intervals for a minimum of 2 years unless study was prematurely terminated because of patient needs for 24-h homecare or institutionalization, withdrawal of consent or death. Follow-up visits included neuropsychological testing, neuropsychiatric evaluations, and MRI. In all included patients, the initial diagnoses were confirmed in the follow-up visits.

Patients with probable AD (McKhann et al. 1984) suffered from progressive memory impairment as presenting symptom which prevailed over other neuropsychological deficits in follow-up visits, confirmed by neuropsychological testing (subtests learning and retention of a word list and recall of geometric figures of the CERADplus neuropsychological battery). Behavioral and neuropsychiatric symptoms, documented by informants using the neuropsychiatric inventory (Cummings 1994), or language impairment was not reported or insignificant. None of the AD patients had a history of fluctuations of cognition or alertness or parkinsonian motor syndrome. Cerebral MRI revealed bilateral atrophies, mainly of the medial temporal lobes and the temporoparietal neocortex, in 3 of 20 patients, albeit to a minor degree, also of the frontal cortex.

Patients with probable FTDbv (Raskovsky et al. Rascovsky et al. 2011) presented with typical progressive behavioral changes and loss of insight confirmed by informants using the NPI (Cummings 1994) and the Frontal Behavioral Inventory (Kertesz et al. 2000), and verified by clinical examination, without language impairment, significant extrapyramidal motor symptoms or psychotic episodes. They exhibited deficits in frontal-executive tasks, had unremarkable visuospatial abilities, relative sparing of short-term memory functions (CERADplus, Schmid et al. 2014, Frontal Assessment Battery, Dubois et al. 2000, Clock Drawing Test, Sunderland et al. 1989, and Stroop Word Colour Test, Stroop 1935). MRI imaging and FDG-PET (in 9 of 21 patients) revealed bilateral frontal and/or temporal lobe atrophies or hypometabolism, respectively, and also medial temporal atrophies.

Patients with Parkinson’s disease dementia (PDD) and Dementia with Lewy Bodies (DLB, *N* = 20) fulfilled the clinical criteria of probable PPD (Emre et al. 2007; Dubois et al. 2007) and probable DLB McKeith et al. 2005), respectively. All patients had a levodopa-responsive Parkinson motor syndrome (Queen’s Square Brain Bank Criteria for PD, and Hughes et al. 1992) and dementia, based on history and clinical and neuropsychological evaluation according to the literature (Dubois et al. 2007; CERADplus, MMSE, Clock Drawing Test).

Demographic, clinical, neuropsychological, and neuropsychiatric data and MRI ratings of subcortical deep white matter lesions (using axial FLAIR images, Fazekas et al. 1993) and medial temporal lobe atrophy (Scheltens et al. 1992) are summarized in Table 1.

**Table 1:** Demographic, clinical, neuropsychological and neuropsychiatric data, MRI ratings of subcortical deep white matter lesions and medial temporal lobe atrophy

	AD (*N* = 20)		FTDbv (*N* = 21)		PDD/DLB (*N* = 20)	
	Mean ± SD	Median	Mean ± SD	Median	Mean ± SD	Median
Age	76.9 ± 6.7	77	75.8 ± 5.7	77	74.8 ± 8.5	77
Sex	11 m, 9f		10 m, 11f		7 m, 13f	
Disease duration	35.6 ± 21.3 months	36	44.3 ± 43.9 months	36	9.7 ± 7.9 years	8 years
MMSE	24 ± 4.1	23.5	23.3 ± 5.1	25	21.8 ± 5.3	22.5
FAB sum score			12.3 ± 4.3	14		
NPI Sum Score	4.7 ± 6.6	2	48.1 ± 30.1	40		
Fazekas Score	0.95 ± 0.88	1	1.23 ± 0.88	1	1.45 ± 1.0 (MRI *N* = 12, CT *N* = 8)	1
Scheltens Score	3.29 ± 0.75	3.5				
Hoehn und Yahr Score					3.4 ± 0.5	3.5

### EEG data acquisition

During EEG recording, patients were awake, and delirium, a complication of dementia, was excluded. The EEG recordings of the AD patients were made using an α-EEG and Neurospeed software (Alpha Trace, Austria). Recordings of the DLBPD and bvFTD patients were made using a Sienna digital EEG (EMS Biomedical, Austria). The EEG amplifiers were automatically calibrated before each recording. Nineteen silver chloride electrodes were placed according to the international 10–20 system. Connected mastoids were used as a reference, and the ground electrode was located in mid-frontopolar position. Horizontal and vertical electro-oculograms (EOG) were recorded from electrodes above and below the left eye and at the outer corners of both eyes. The electrocardiogram (ECG) was acquired using a wrist clip electrode. The signals were amplified, bandpass (0.3–70 Hz) and notch (50 Hz) filtered, and digitized at 256 Hz with a resolution of 16 bits. Impedances were kept below 10 kΩ. The recordings were made in a quiet room, with patients in a sitting or lying position. They were awake with eyes closed. Patients were instructed to avoid movements of the eyes, body, or facial musculature, all monitored by clinicians.

### EEG pre-processing

Before computing quantitative features, the following pre-processing steps were performed. (1) The first 20 s of continuous artifact-free recording were selected using visual inspection by an experienced expert and confirmed by a second expert. Consequently, sections with artifacts caused by poor electrode contacts or patient movement/talking that appeared as excessive voltages were excluded from further assessment. (2) Artifact produced by eye movements and blinks was eliminated by linear regression using the horizontal and vertical EOG signals. (3) Interference caused by the electric field of the heartbeat was picked up by the EEG leads and detected by the high-impedance EEG amplifier. This form of interference was corrected for using a modified Pan-Tompkins algorithm and linear regression (Waser and Garn 2013). (4) A 2-Hz digital high-pass filter with finite impulse response was used to eliminate fluctuations of the measured voltage caused by sweating. (5) Finally, a sliding window was moved automatically over the artifact-free, interference-corrected sections of the EEG to determine a series of 4-s epochs with an overlap of 2 s. These epochs were used to compute the QEEG features.

### QEEG features

Twenty-five QEEG features were applied in this study: relative band powers, spectral ratios, center frequency, auto-mutual information, cross-mutual information, coherences, phase coherences, partial coherences, Granger causality (GC), and conditional GC.

Frequency (spectral) measures: Frequency measures seemed relevant for this study because shifts in relative band power have been observed with many neurodegenerative disorders, particularly with AD. This phenomenon has frequently been described as the “slowing” of the EEG (Jeong 2004). We computed relative band powers in the *δ* (2–4 Hz), *θ* (4–8 Hz), *α* (8–13 Hz), and *β*1 (13–20 Hz) frequency bands for each of the selected epochs using an indirect spectral estimator (Waser et al. 2016). Values for each frequency band were expressed as a percentage of the power in the total 2–20 Hz range. Spectral ratios are the ratios of low-frequency band power over high-frequency band power: *R*1 = *θ*/(*α*+*β*1); *R*2 = (*δ* + *θ*)/(*α* + *β*1); *R*3 = *θ*/*α*; *R*4 = *θ*/*β*1. Center frequency was computed as the center of gravity of the 2–20 Hz spectrum. In addition, relative global field power was determined for each of the four frequency bands. Relative global field power is the ratio of the absolute power of a frequency band from all electrodes over the absolute power of the total frequency band (Nishida et al. 2011).

Synchrony measures: The term synchrony refers to the degree of simultaneousness of the brain waves measured at different locations over the cortex. Changes in synchrony have been reported to be associated with functional disconnections among cortical areas, which might be caused by the death of cortical neurons, axonal pathology, or cholinergic deficits. A decrease in the coherence of fast EEG rhythms has been described as the hallmark of EEG changes in AD (Jeong 2004). Coherence between two channels describes the degree of association of the frequency spectrum between two referential or bipolar signals (Rosenberg et al. 1989), which can be applied to both the signals’ amplitude and phase. Partial coherence measures only direct spectral dependencies between the two signals. Amplitude and phase coherence as well as partial coherence were computed in the four frequency bands of *δ*, *θ*, *α*, and *β*1 specified above. GC (Granger 1969) describes whether the time course of the EEG in channel X can help to predict the future values of the EEG signal in channel Y. Conditional GC measures analogously only direct synchrony between channel X and channel Y (Flamm et al. 2012). GC and conditional GC were computed in the frequency range of 2–20 Hz and in both directions (e.g., F4/P4 and P4/F4).

Measures of information theory: information processing in the brain is a highly nonlinear, dynamic procedure. Electric potentials measured by EEG are generated by nonlinear coupling interactions between neuronal populations, performing information transfer. Such activities can best be characterized using information-theoretic measures (Jeong 2004). Auto-mutual information measures the mutual dependence of the EEG signal and its time-shifted version in the same channel (Cover and Thomas 1991). Cross-mutual information measures the mutual dependence of a time signal and the time-shifted version of the signal in another channel. Thereby, cross-mutual information describes the synchrony between two different regions of the cortex in the time domain. Both measures were computed in the frequency range of 2–20 Hz.

All computations were performed using Matlab R2013b. Values were averaged over 20-s periods.

### Statistical analyses

#### QEEG features in relation to MMSE scores

Multiple linear regression was used to determine whether the QEEG features could be explained by the MMSE scores. For this study, apart from the MMSE scores, the demographics were introduced as co-variables in linear and quadratic terms. Thus, the x-predictors for the linear regression were the following: MMSE, MMSE^2^, age, age^2^, and gender.

The coefficients of determination (*R* ^2^) describe how well the model predicts the y-response in terms of the x-predictors, in proportion to the amount of variation of the y-response. The *p* values for Fisher’s *F* tests indicate the models’ significance. Scatter plots of the QEEG features of each patient group were drawn for visual inspection. Multiple linear regressions were computed with MMSE scores as the x-predictors and the QEEG feature variables as y-response.

Among the multitude of significant regressions, features with opposite slopes between pairs of patients groups were of interest in this study, because these could be clues for supporting differential diagnoses.

#### Dementia group comparisons (Mann–Whitney *U* test)

In addition, the dementia groups were compared using Mann–Whitney *U* test. This is a nonparametric test for equality of population medians between two independent samples. *U* tests were applied to determine if any of the QEEG features showed significant differences between the groups. Mann–Whitney *U* test was performed between AD versus DLBPD, AD versus DLBPD, and bvFTD versus DLBPD. A *p* value of <0.05/25 = 0.002, Bonferroni corrected for 25 features, was considered significant.

#### Classification between dementia groups

Support vector machine (SVM) is a classification method for data consisting of two classes. It classifies by searching the best hyperplane that separates all data points of the two classes. The best hyperplane is the hyperplane with the largest maximal width of the slab parallel to the hyperplane that has no interior data points. In this case, the QEEG features are the data in which the SVM method searches for a division in the data to form two classes. The SVM classifiers classify each of the 61 patients to a dementia group using the QEEG features. The correctness of the classifications was then validated by the real clinical diagnoses of the patients.

The selection of QEEG features is crucial to the performance of an SVM classifier. It is important to choose features that would distinguish between the dementia groups. As such, the classification process consisted of feature reduction and selection to achieve a classifier with a minimal misclassification rate.

#### Feature reduction for classification

Classification of the patients was performed based on referential EEG data. A total of 25 QEEG features were computed on all electrode or electrode channel pairs and, when applicable, on the corresponding frequency bands. Not all these features could be expected to contribute to the classification of the groups. Therefore, feature reduction by Mann–Whitney *U* test and principal component analysis (PCA) was employed to alleviate the computational burden, to reduce complexity of computation, and to remove nonfactor variables. The reduction of features was done by eliminating QEEG features that exhibited a significance level in difference of *p* < 0.05/25 in Mann–Whitney *U* test.

PCA transforms data into new variables called principal components. Each of these principal components is a linear combination of the original variables that all components are orthogonal to each other to avoid redundancy of data. With PCA, feature variables were selected depending on the following criteria:

1. total variance in the principal components up to 75/80/85/90/95%;
2. threshold for coefficients (loadings) 0.15/0.20/(0.30 for three-way classification only).

For example, if a 90% total variance was considered with >0.20 absolute loadings value, then only the principal components amounting to 90% total variance were chosen. All variables from the chosen principal components with loadings >|0.20| value are selected.

Ten different combinations of total variance and loadings absolute value were used in this study as the initial training data for the next part of the classification process. Included as well were the features selected through the Mann–Whitney *U* test.

#### Classification between two dementia groups

Classification between two groups involved feature selection and SVM classification with leave-one-out cross validation. This classification was done for AD versus DLBPD, AD versus bvFTD, and bvFTD versus DLBPD.

A wrapper method type of feature selection was employed by means of a reverse sequential feature selection. This procedure used the resulting features from the feature reduction process as the initial training data set. In every step, it then proceeded to combine and evaluate different subsets of features by means of an SVM classification model with leave-one-out cross validation. It then eliminated one variable by each step to improve the misclassification rate. The procedure stopped once it reached a point where features could no longer be eliminated to improve the misclassification rate. At this point, all the remaining features in the training data set were considered as the final set of training data, giving the optimum accuracy rating. The results of this process yielded the best training data set for the classification of two dementia groups.

#### Three-way classification

This algorithm used the classifiers from classification between two groups. It consisted of two parts: (1) classify whether group A or not and (2), if not, classify whether group B or C. This resulted in two sets of feature variables as training data for classification. For example, the first part is a classification between AD and not AD. If it classifies as AD, the algorithm classifies it as AD; if not, then it classifies between DLBPD and bvFTD.

#### Validation of classifiers

The “leave-one-out” method validates the classifiers within one and the same patient group: the classifier is trained with, for example, the first 60 patients and the 61st patient is classified. Then, the first 59 patients and the 61st patient are used for training and the 60th is classified, and so on. The overall accuracy is then determined based on all 61 classification results.

## Results

### QEEG features in relation to MMSE scores

**Fig. 1:**
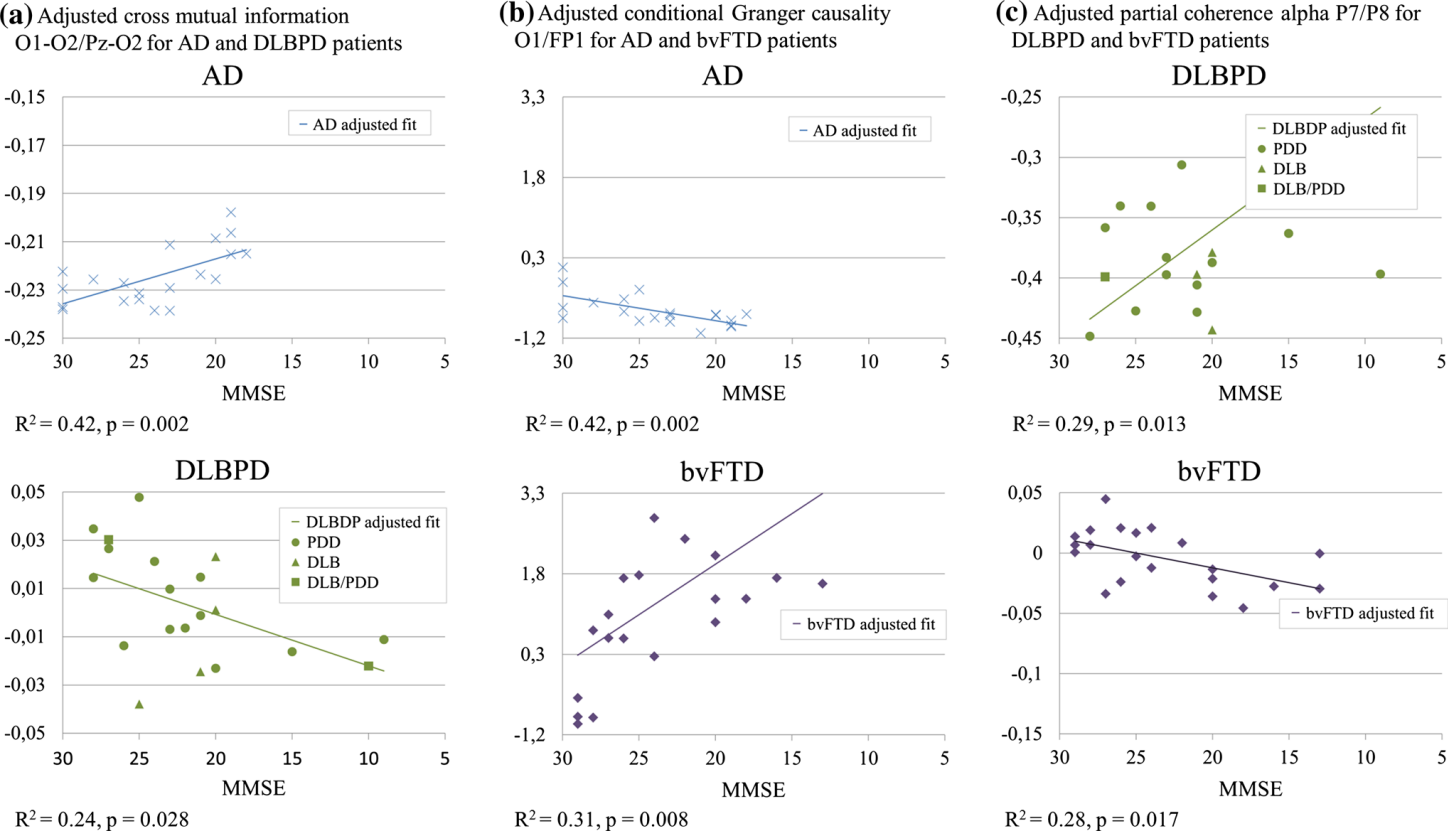
Significant regression models

AD versus DLBPD: Opposite slope trends (increase with decreasing MMSE in AD and increase with increasing MMSE in DLBPD) were evident for features relative β1 band power Pz–O2, auto-mutual information Pz–O2, and cross-mutual information O1–O2/Pz–O2. The electrode sites Pz and O2 are of particular note as both were involved in several features (opposite trends of the AD and DLBPD groups in relation to MMSE scores for auto-mutual information, relative band power β1, and cross-mutual information). As an example, cross-mutual information is shown in Fig. 1a.

AD versus bvFTD: Opposite slope trends were evident for conditional GC Fp1/Fp2, where it decreases as the MMSE score decreases in bvFTD patients and it increases as the MMSE score decreases in AD patients. Trends in opposite directions were also observed in GC O1/Fp1 (Fig. 1b).

bvFTD versus DLBPD: Opposite slope trends were evident in partial α coherence in P7/P8 which increases as the MMSE score decreases in DLBPD patients and decreases as the MMSE score decreases in bvFTD patients (Fig. 1c).

### Dementia group comparisons (Mann–Whitney *U* test)

The Mann–Whitney *U* test was performed for all QEEG features from referential data. Considering the multiple tests performed on the data sets, the strict criterion of Bonferroni for statistical significance was applied (*p* < 0.002).

**Fig. 2:**
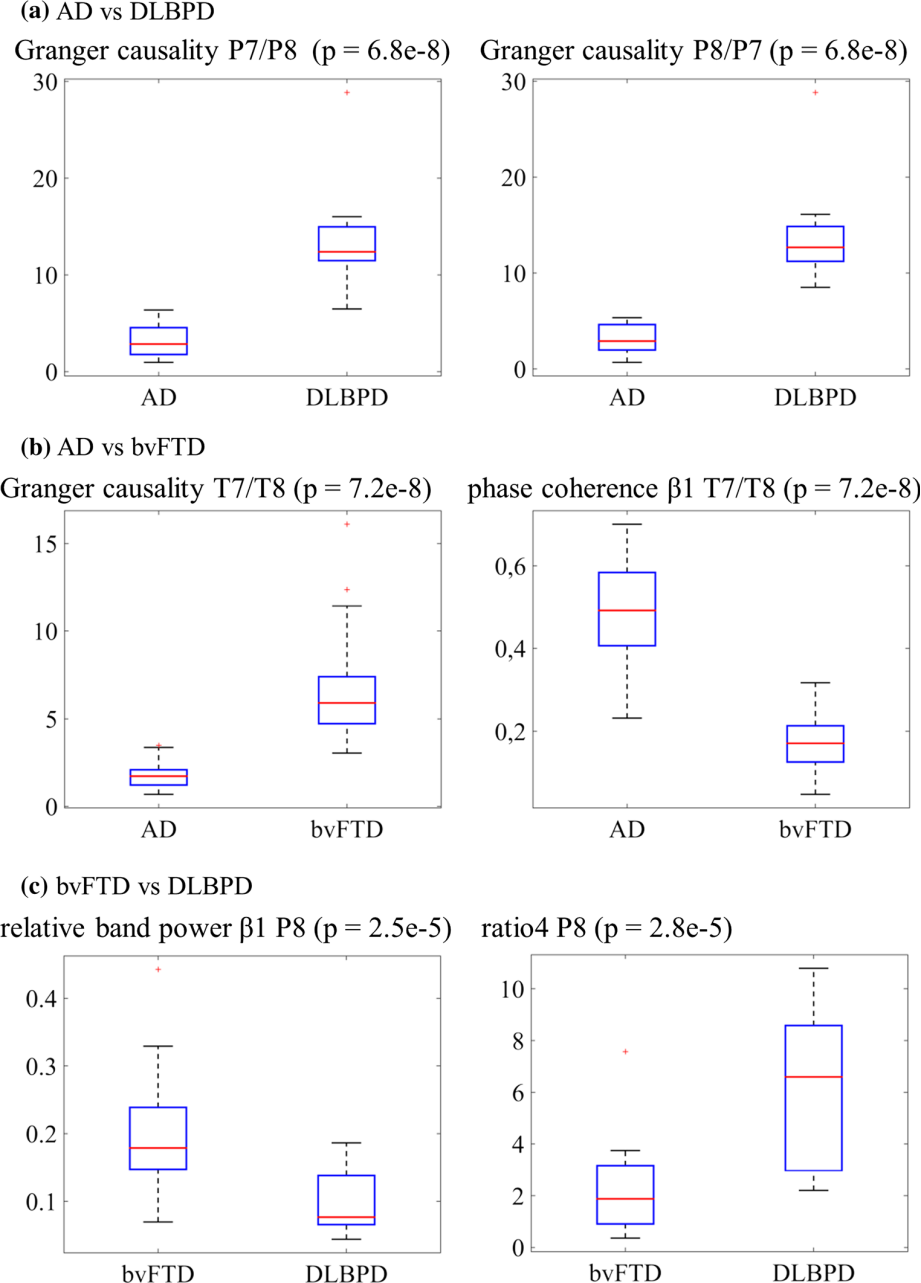
Boxplots of selected features

AD versus DLBPD: 23 of 25 features resulted in significant differences. The features with the lowest *p* values (*p* = 6.80e-08) were the Granger causalities at P7/P8 and P8/P7 (see Fig. 2a). Center frequency and relative band power α and β1 were higher in AD patients than in DLBPD patients at all sites with significantly different results. The opposite was true for auto-mutual information, band ratios, relative band power θ, and cross-mutual information.

AD versus bvFTD: 17 features resulted in significant differences with GC and phase coherence β1 reaching the lowest *p* value of 7.21e-8 at T7/T8. GC was significantly higher in bvFTD patients than in AD patients while phase coherence was significantly higher in AD patients (see Fig. 2b). Phase coherence α and β1 was significantly higher in AD patients than in bvFTD patients at all sites with significantly different results. The opposite was observed for coherence β1 and GC.

bvFTD versus DLBPD: 12 features resulted in significant differences with relative band power β1 at P8 and ratio4 at P8 having the lowest *p* value of 2.53e-5 and 2.83e-5, respectively. Auto-mutual information and mutual information were higher in DLBPD patients than in bvFTD patients at all sites with significantly different results. The opposite was then evident for center frequency.

### Classification results

#### Classification between two dementia groups

Classifications between AD and DLBPD, AD versus bvFTD, and bvFTD versus DLBPD resulted in 0% misclassification rate (100% accuracy) based on leave-one-out validation. With the number of patients limited to 20 or 21 patients per dementia group, the steps of accuracy are 2.5 and 2.4%. GC and ratio 4 (R4) were used as features for training of the classifiers. The electrodes sites chosen as features for each classifier did not necessarily follow the results of the Mann–Whitney *U* Test. These results were expected as the two did not follow the same mathematical model. The selection of features based from the sequential feature selection highlighted combination of features that would give the best classifier results. Most selective electrode sites for each classifier are thus:

AD versus DLBPD: GC: P8/P4; R4: O1;

AD versus bvFTD: GC: Fp1/F7, C3/P7, P3/Fp1, F3/Fp1, T8/F8; R4: P7, Pz;

bvFTD versus DLBPD: GC: F8/T8, P4/O2, P4/C4, F8/F7, F4/F3, C4/C3, P8/P7, C3/F7, C3/F3, C4/F4, O2/P8, P8/P4; R4: Pz, O1.

#### Classification between one dementia group and the rest

**Fig. 3:**
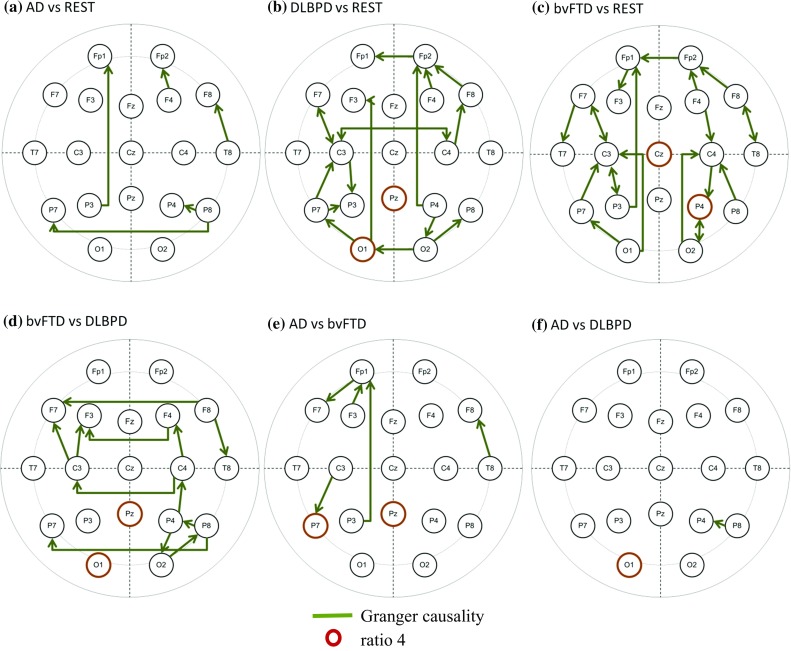
Optimal features and electrode sites or pairs of electrodes for classification

Again, 100% accuracy was achieved for all three classifiers. Distinguishing between AD and the rest was possible based on just one feature (i.e., GC). The classification of DLBPD versus the rest required the features GC and R4. The same two features were also used for differentiating bvFTD to achieve the same accuracy. Most selective electrode sites or electrode pairs are:

AD versus rest: GC: P3/Fp1, P8/P7, F4/Fp2, T8/F8, P8/P4 (Fig. 3a);

bvFTD versus rest: GC: Fp1/F3, F7/C3, F7/T7, F8/T8, F4/C4, P4/O2, P3/C3, P3/Fp1, O1/C3, O2/C4, Fp2/Fp1, F8/Fp2, F4/Fp2, C3/F7, T8/F8, O1/P7, O2/P4, P7/C3, P4/C4, C3/P3, C4/P4; R4: Cz, P4 (Fig. 3b);

DLBPD versus rest: GC: C3/C4, F7/C3, P4/O2, P4/Fp2, O1/F3, Fp2/Fp1, C4/C3, O2/O1, F8/Fp2, F4/Fp2, C3/F7, C4/F8, O1/P7, O2/P8, P7/C3, C3/P3, P7/P3; R4: Pz, O1 (Fig. 3c).

#### Combination of SVM classifiers

Three different combinations of SVM classifiers were tested for maximum overall accuracy:

1. AD versus REST then bvFTD versus DLBPD;
2. DLBPD versus REST then AD versus bvFTD;
3. bvFTD versus REST then AD versus DLBPD;

The first combination (1) did the job with the least number of features and electrode sites (Fig. 3a and d):

1. SVM 1: GC: P3/Fp1, P8/P7, F4/Fp2, T8/F8, P8/P4;
2. SVM 2: GC: F8/T8, P4/O2, P4/C4, F8/F7, F4/F3, C4/C3, P8/P7, C3/F7, C3/F3, C4/F4, O2/P8, P8/P4; R4: Pz, O1.

## Discussion and conclusions

Optimal features for differential diagnosis were found by applying extensive signal processing: Pre-processing steps reduced the influence of artifacts, such as eye movements, blinking, and heartbeat. An advanced method for estimating signal spectra was applied. Our study investigated 25 QEEG features characterizing frequency (spectral) properties, synchrony, and similarity of signals. For analysis, scatter plots of these QEEG features versus MMSE scores were generated with linear regression lines with age and sex introduced as co-variables. Using the Mann–Whitney *U* test, differences in QEEG features among the three dementia groups were established. Finally, SVM classifiers were trained with a selection of features to form a tool for differentiating the dementia groups from each other.

Slowing of the frequency spectrum, which has long been known to be a hallmark in dementia, turned out to represent one of the two most significant features for differential diagnosis. This is well in line with the results of the previous scientific studies (Jeong 2004). For enhancing accuracy, GC has been introduced for the first time and added as an additional feature for classifier training.

Another novelty of our study is the investigation of the relationships of QEEG features to severity of the diseases measured in MMSE scores: In plotting QEEG features versus the MMSE scores, auto-mutual information Pz–O2 and cross-mutual information O1–O2/Pz–O2 were found to increase as the MMSE score decreases in AD patients and it decreases as the MMSE decreases in DLBPD patients. The opposite was observed for relative β1 band power Pz–O2. Comparing AD and bvFTD, conditional GC Fp1/Fp2 increases as the MMSE score decreases in AD patients and it decreases as MMSE score decreases in bvFTD patients. The opposite was observed for conditional GC O1/Fp1. A difference was observed for the feature partial coherence α at P7/P8 between bvFTD and DLBPD patients, where there was an increasing trend in DLBPD as the MMSE scores decreased and the opposite for bvFTD.

The Mann–Whitney *U* test results showed that center frequency, relative band power α, and relative band power β1 at 14, 15, and 13 electrode sites, respectively, were found to be significantly different with *p* < 0.002 between AD and DLBPD patients. GC, phase coherence α, phase coherence β1, and coherence β1 features at 29, 14, 16, and 18 sites or pairs of sites, respectively, were significantly different for the differentiation between AD and bvFTD. Auto-mutual information, mutual information, and center frequency at 5, 6, and 5 sites or pairs of sites, respectively, for differentiating bvFTD and DLBPD were found to be significant. This finding demonstrates AD and DLBPD to be most dissimilar based on the number of electrode sites results of the Mann–Whitney *U* test. Certain features, such as auto-mutual information, band ratios, relative band power *θ*, and mutual information, were consistently higher in DLBPD patients than in AD patients given a significant difference between the two. The opposite was also true for the features center frequency and relative band power *α* and *β*1. Comparing AD and bvFTD, it was observed that phase coherence *α* and *β*1 were higher in AD patients than in bvFTD patients. The opposite was observed for coherence *β*1 and GC. Auto-mutual information and cross-mutual information were higher in DLBPD patients than in bvFTD patients, whereas center frequency is higher in bvFTD patients than in DLBPD patients.

SVM classifiers were obtained by reducing the number of features using PCA and Mann–Whitney *U* test. Feature selection was achieved using backwards sequential selection.

Classifiers between two dementia groups and between one dementia group and the rest performed well with 100% accuracy based on leave-one-out validation, with GC and the ratio of high- and low-frequency power as training variables. The selection of the optimal electrode sites and pairs of electrodes was different for each classifier. Although the selection of GC electrode sites did not have any observable pattern or common configuration, the electrode sites for R4 were concentrated in the posterior area (P3, P4, P7, and Pz) and at the occipital lobe (O1). The number of features needed for the classification among AD, DLBPD, and bvFTD with 100% accuracy was two overall. However, it must be noted that, due to the number of patients, accuracy ratings are in the steps of 2.5% for 20 patients (AD versus DLBPD) and 2.4% for 21 patients (bvFTD versus DLBPD and AD versus bvFTD).

This study has indicated that QEEG features might have the potential to support the differentiation between the dementia groups. It has shown that the particular QEEG features GC and the power ratio between the θ and β1 bands (R4), in posterior and occipital lobes, could be of particular benefit in differentiating the diseases.

This study only worked on 61 patients (20 AD, 20 DLBPD, and 21 bvFTD). Thus, it is necessary to conduct further studies with more patient data per group to confirm the results. It is also of interest to add comparisons to normal health controls, although it would not influence the differentiation among AD, DLBPD, and bvFTD patients with MMSE scores less than 30.

Patients fulfilled the current clinical criteria of probable AD, FTDbv, and DLB/PDD. The diagnoses were verified in clinical follow-up examinations, comprising in AD and FTDbv patients also follow-up MRI, neuropsychological testing, and structured neuropsychiatric evaluation. CSF analysis for tau, phosphorylated tau, and Aß1-42 could have contributed to higher diagnostic accuracy, especially in the differential diagnosis between FTDbv and AD, even though there are still uncertainties about how to interprete methodological variabilities and to overlap in the CSF findings between these two diseases (Leuzy et al. 2016). Amyloid PET might have added to higher diagnostic probability in our study. However, diagnostic specificity of amyloid PET in AD does not exceed 90% and false-positive findings or mixed pathologies may occur in FTDbv (Clark et al. 2012; Curtis et al. 2015; Rabinovici et al. 2011; Sabri et al. 2015).

There were nine previous studies that investigated differences in QEEG features among patients with AD, patients with DLB or PDD, and patients with FTD. The authors used EEG band powers, coherences, dominant frequencies, peak α frequencies, and cortical sources. Only three of these papers reported quantitative classification results: Snaedal et al. (2012) also used an SVM for classification and achieved 91% accuracy in differentiating AD from DLBPD, 93% for DLBPD-FTD and 88% for AD-FTD (239 AD, 52 DLBPD, and 14 FTD patients). The authors used *θ*, *α*2, and *β*1 coherences together with peak α frequency for classification. Andersson (Andersson et al. 2008) evaluated EEG variability in dementia with DLB and AD. The DLB group showed higher overall coherence in the delta band and a lower overall coherence in the alpha band than the AD group. These features distinguished the two patient groups with areas under the ROC curves between 0.75 and 0.80 and 0.91 and 0.97, respectively. Caso et al. (2012) differentiated AD from FTD patients via 12 spectral parameters in the delta, theta, alpha 1 and 2, and beta 1, 2, and 3 frequency bands, achieving 49% sensitivity and 85% specificity (39 FTD and 39 AD patients). Lindau et al. (2003) differentiated AD from FTD patients as well, achieving 93.3% classification accuracy (19 FTD and 16 AD patients). They used *δ* and *θ* activities together with visuospatial ability and episodic memory.

Most studies on the differentiation among AD, DLB or PDD, and FTD were done using SPECT or, in one case, PET and, in another case, MR. Some of these studies were analyzed in the recent review by Brigo et al. (2015). For differentiating AD from DLB or PDD patients by SPECT, sensitivities and specificities in the range of 46 up to 100% were found (Lobotesis et al. 2011; O´Brian et al. O’Brien et al. 2004; Colloby et al. 2004; Hanyu et al. 2006; McKeith et al. 2007; Colloby et al. 2008; Morgan et al. 2012; Brockhuis et al. 2015). Using PET, Gilman (Gilman et al. 2005) reported 64.3% sensitivity and up to 69.6% specificity for classifying DLB. Horn et al. (2007) and Stühler et al. (2011) differentiated AD and FTD patients by SPECT with sensitivities and specificities in the range of 82–87.5%. Spehl et al. (2015) included FTD, DLB, and AD patients. Differentiation between the groups reached areas under the receiver-operating curve between 0.74 and 0.97. The highest accuracy in differentiation was reached for DLB and AD and the lowest for FTD and AD. Zhong (Zhong et al. 2014) used ^1^H-proton MR spectroscopy to differentiate DLB from AD. For 19 DLB and 21 AD patients, the mean areas under the receiver-operating characteristics (ROC) curves of glutamate concentrations in the occipital lobe were 0.773 (with 66.7% sensitivity and 84.2% specificity). Franciotti et al. (2013) compared default mode networks AD and DLB patients and found that functional connectivity was reduced in these patients compared to controls. Posterior cingulate cortex activity was lower in AD than in control subjects and DLB patients. The functional connectivity in the right hemisphere was reduced in DLB patients in comparison with controls, but not in AD patients.

Due to the complexity of the brain, we are not able to specify direct interrelations among electrophysiological, structural, and metabolic markers today. However, parallels can be found: in MRI, diffusion-tensor imaging (DTI), fMRI, SPECT, and PET studies, differences between AD and DLBPD groups were predominantly found in parieto-occipital and frontal and, to a lesser degree, in temporoparietal electrodes/electrode combinations, in our study in parieto-occipital locations. bvFTD is characterized by frontal and temporal cortical and subcortical atrophy, whereas posterior cortical areas, including the posterior parietal and the occipital lobes, are preserved, which differs from AD and DLBPD (Binnewijzend et al. 2014; Vemuri et al. 2011) with sensitivities of MRI atrophy pattern recognition ranging between 78 and 90% and specificities of 84–98%. In our study, differences of DLBPD or AD to bvFTD were observed largely in temporoparietal locations.

Compared to these results from SPECT, PET, and MR, our results from QEEG seem to provide similar or even higher accuracy, sensitivity, and specificity. This was shown in this paper using comparable numbers of subjects. The major difference between SPECT/PET and EEG is that the EEG is non-invasive, much simpler to perform, widely available, and cost-effective.

We conclude that classifiers trained with selected QEEG features might have the potential to provide another valuable means of supporting the differentiation between AD, DLBPD, and bvFTD patients and that QEEG might offer substantial advantages over other biomarkers in terms of practicability and accuracy. Future studies should test this hypothesis for application not only on group level but also for individual subjects using larger numbers of patients.
